# Supplementary material for: SCFFbxw5 targets kinesin‐13 proteins to facilitate ciliogenesis
Source: EMBO J. 2021 Aug 9;40(18):e107735. doi: 10.15252/embj.2021107735 (PMC8441365; doi:10.15252/embj.2021107735)
Supplement: Supplementary file 2 — Appendix [file EMBJ-40-e107735-s001.pdf]

Appendix file – Schweiggert *et al.*

Table of Contents

Appendix Table S1: Plasmids. 2
Appendix Table S2: Reagents 6

Appendix Table S1: Plasmids.

Plasmid	Template for PCR/Method	Oligonucleotides	Source
pET22B-Uba1-His			[24]
pGEX6P3-GST-Cdc34			[24]
pGEX6P3-GST-UbcH5b			[24]
pGEX6P3-GST-UbcH5c			A. Pichler
pGEX6P3-GST-Ube2G1	cDNA of RPE-1 cells (Gibson)	5'-CCTGGGATCCCCGAATTCCATGACGGAGCTGCAGTC-3'<br>5'-TGCGGCCGCTCGAGTCGACTCACTCAAAAGCAGTCTCTTGG-3'	This work
pGEX-GST-Nedd8			B. Schulman
pGEX-GST-Ubc12			B. Schulman
pGEX-GST-APPBP1-rbs-Uba3			B. Schulman
pGEX-4T-GST-PreScission			A. Dickmanns
pET28a-His-Ubiquitin			[24]
pET3a-Ubiquitin			S. Raasi[69]
pET3a-Ubiquitin(K48R)	pET3a-Ubiquitin (mutagenesis)	5'-TTGATCTTTGCCGGTAgGCAGCTCGAGGACGGTA-3'<br>5'-TACCGTCCTCGAGCTGCcTACCGGCAAAGATCAA-3'	This work
pET3a-Ubiquitin(K63R)	pET3a-Ubiquitin		S. Raasi[69]
pET3a-Ubiquitin(K48R/K63R)	pET3a-Ubiquitin(K63R) (mutagenesis)	5'-TTGATCTTTGCCGGTAgGCAGCTCGAGGACGGTA-3'<br>5'-TACCGTCCTCGAGCTGCcTACCGGCAAAGATCAA-3'	This work
pENTR221-GSTP1 (clone ID 182399431)			DKFZ ORF Collection
pENTR221-CSRP2 (clone ID 176815824)			DKFZ ORF Collection
pENTR221-Sec23B (clone ID 128836251)			DKFZ ORF Collection
pENTR221-HGS (clone ID 158422822)			DKFZ ORF Collection
pENTR223-CSPP1 (clone ID 148371129)			DKFZ ORF Collection
pENTR221-ODF2 (clone ID: 142932633)			DKFZ ORF Collection

pENTR221-Kif2C (clone ID 199650980)			DKFZ ORF Collection
pENTR221-Kif2A (clone ID 140305080)			DKFZ ORF Collection
pENTR223-Kif2B (clone ID 123156095)			DKFZ ORF Collection
pcDNA3.1-HA-GSTP1	pENTR221-GSTP1 (XhoI/KpnI)	5'-CGGCTCGAGATGCCGCCCTACACCGTG-3'<br>5'-CGGGGTACCCTACTGTTTCCCGTTGCC-3'	This work
pcDNA3.1-HA-CSRP2	pENTR221-CSRP2 (XhoI/KpnI)	5'-CGGCTCGAGATGCCTGTCTGGGGAGGTG-3'<br>5'-CGGGGTACCTTACTGGGCATGAACAAGAGC-3'	This work
pcDNA3.1-HA-Sec23b	pENTR221-Sec23B (XhoI/KpnI)	5'-CGGCTCGAGATGGCGACATACCTGGAG-3'<br>5'-CGCGGTACCTTAACAGGCACTGGAGACAGC-3'	This work
pcDNA3.1-HA-HGS	pENTR221-HGS (XhoI/KpnI)	5'-CGGCTCGAGATGGGGCGAGGCAGCGGC-3'<br>5'-CGGGGTACCTCAGTCGAATGAAATGAGCTG-3'	This work
pcDNA3.1-HA-CSPP1	pENTR223-CSPP1 (XhoI/HindIII)	5'-CGGCTCGAGATGAGACAGCCTTCTCCTATAG-3'<br>5'-CGGAAGCTTTTAACCATGTGCAGTCGACAG-3'	This work
pcGS5-HA-DMTN			P. Sauer
pcDNA3.1-HA-ODF2	pENTR221-ODF2 (BamHI/XbaI)	5'-CGGGGATCCATGAAGGACCGCTCTTCAACTC-3'<br>5'-CGGTCTAGATCAGGCAGGAGGAGATCGGGAG-3'	This work
pcDNA3.1-HA-MCAK	pENTR221-Kif2C (BamHI/NotI)	5'-ATATGGATCCATGGCCATGGACTCGTCG-3'<br>5'-TATAGCGGCCGCCTACTGGGGCCGTTTCTTG-3'	This work
pcDNA3.1-HA-Kif2A	pENTR221-Kif2A (BamHI/NotI)	5'-TATAGGATCCATGGTAACATCTTTAAATG-3'<br>5'-TATAGCGGCCGCTTAAAGGGCACGGGGTC-3'	This work
pcDNA3.1-HA-Kif2B	pENTR223-Kif2B (EcoRI/NotI)	5'-TATAGAATTCATGGCCAGCCAGTTC-3'<br>5'-TATAGCGGCCGCTCACTCTACCTTGCTC-3'	This work
pcDNA3.1-Flag			[24]
pcDNA3.1-Flag-Fbxw5			[24]
pcDNA3.1-Flag-Fbxw5-ΔF-box			[24]
pCMV-3xFLAG-Fbxw7			I. Hoffmann[77]
pET28a-His-MCAK	pcDNA3.1-HA-MCAK (BamHI/NotI)	5'-ATATGGATCCATGGCCATGGACTCGTCG-3'<br>5'-TATAGCGGCCGCCTACTGGGGCCGTTTCTTG-3'	This work
pET28a-His-Kif2A	pcDNA3.1-HA-Kif2A (BamHI/NotI)	Cloned by restriction digestion	This work
pET28a-His-Kif2B	pcDNA3.1-HA-Kif2B	Cloned by restriction digestion	This work

	(EcoRI/NotI)		
pFastBacHT-B-His-Fbxw5			[24]
pFastBac1-Skp1ΔΔ			[24,43]
pFastBacHT-B-Eps8	pCDNA3.1-HA-Eps8[24]	5'-GTATTTTCAGGGCGCCATGGATGAATGGTCATATTTCTAATCATC-3'<br>5'-CAGGCTCTAGATTCGAAAGCTTAGTGACTGCTTCCTTC-3'	This work
pFastBacHT-B-MCAK	pcDNA3.1-HA-MCAK (BamHI/NotI)	5'-ATATGGATCCATGGCCATGGACTCGTCG-3'<br>5'-TATAGCGGCCGCCTACTGGGGCCGTTTCTTG-3'	This work
pFastBacHT-B-His-Kif2A	pcDNA3.1-HA-Kif2A (BamHI/NotI)	Cloned by restriction digestion	This work
pEGFP-PLK1-T210D			S. Lev
pFastBacHT-B-His-Plk1-T210D	pEGFP-PLK1-T210D (Gibson)	5'-AACCTGTATTTTCAGGGCGCAATGAGTGCTGCAGTGACTG-3'<br>5'-CAGGCTCTAGATTCGAAAGCTTAGGAGGCCTTGAGACG-3'	This work
pcDNA3.1-Flag-Fbxl2			L. Hengst
pFastBacHT-B-His-Fbxl2	pcDNA3.1-Flag-Fbxl2	5'-GTATTTTCAGGGCGCCATGGTTTTCTCAAACAATGATGAAGG-3'<br>5'-CTCTAGATTCGAAAGCGGCCTCAGAGAATGACACAGCAC-3'	This work
pFastBacHT-B-His-Fbxw7	pCMV-3xFLAG-Fbxw7		This work
pET11D-hRanGAP			A. Flotho
pBIG1B-His-Cul4A/His-DDB1/His-mmRbx1			E. Fischer
pBIG1B-His-Cul4A/His-DDB1/mmRbx1	pBigBac-His-Cul4A/His-DDB1/His-mmRbx1 (Gibson, 4 fragments)	5'-ATCTACCATGGGCACCAACAGC-3'<br>5'-TGACTCGCTGCGCTCGGTCGTTCGGCTGCGGCGAGCGG-3'<br>5'-CGACCGAGCGCAGCGAGT-3'<br>5'-ATGGGCTTGTAGGCGAGCAG-3'<br>5'-TGCTCGCCTACAAGCCCATGG-3'<br>5'-GTCCGGGGGTACCTCATCAGGCCACGTAGTGGTAC-3'<br>5'-CTGATGAGGTACCCCCGGAC-3'<br>5'-TGTTGGTGCCCATGGTAGATCCGTATTTATAG-3'	This work
pBIG1B-HisCul1/mmRbx1	cDNA of RPE-1 (Gibson)<br>destination vector: pBigBac-His-Cul4A/His-DDB1/mmRbx1 cut with SalI/PvuII	5'-CCATCATCATCATCATCACTCCGCGGTCGACGAGAACCTCTACTTC CAGGGAGGCGGCCGCTCGTCAACCCGGAGCCAG-3'<br>5'-GGCTTTACTGGAAATTTCAGTTAAGCCAAGTAACTGTAGGTGTCC-3'	This work
pRetroX-TRE3G-mNG-MCAK			S. Hata

pRetroX-TRE3G-mNG-Fbxw5	mNG tag from pRetroX-TRE3G-mNG-MCAK via Gibson into pcDNA3.1-Flag-Fbxw5 cut with HindIII and BamHI. mNG-Fbxw5 then subcloned via BamHI/NotI into pRetroX	5’-ACTCACTATAGGGAGACCCAATGGTGAGCAAGGGCGAGG-3’<br>5’-GCGTGCCGCCCTCGTCCATGCCGCCTGCACCAGCTCCT-3’	This work
pRetroX-TRE3G-mNG-Kif2B	pcDNA3.1-HA-Kif2B (Gibson) destination vector: pRetro-TRE3G-mNG-MCAK cut with EcorI/NotI	5’-GTGCAGGAGCTGGTGCAGGCATGGCCAGCCAGTTCTGC-3’<br>5’-GCGCCTCCCCTACCCGGTAGTCACTCTACCTTGCTCTTCACG-3’	This work
pQCXIZ-H2B-mRuby2			S. Hata
pRetroX-Tet3G			Clontech®
pVSV-G			Clontech®
p53-HaloTag® Fusion Vector			Promega
NanoLuc®-MDM2 Fusion Vector			Promega
pFN21A-HT-Fbxw5			Promega
pFC32K-MCAK-NLuc	pcDNA3.1-HA-MCAK	5’-GCGTGCGATCGCCATGGCCATGGACTCGTCGCT-3’<br>5’-GGCGGTTTAAACCTGGGGCCGTTTCTTGCTGCT-3’	This work
pFN31K-NLuc-MCAK	pcDNA3.1-HA-MCAK	5’-GCGTGCGATCGCCATGGCCATGGACTCGTCGCT-3’<br>5’-GGCGGTTTAAACCTGGGGCCGTTTCTTGCTGCT-3’	This work

Appendix Table S2: Reagents

**Antibodies**			
**Antibody**	**Species**	**Source**	**Identifier**
Monoclonal anti HA.11	Mouse	Covance	MMS-101R
Monoclonal anti-Flag	Mouse	Sigma Aldrich	F1804
Monoclonal anti-Flag	Rabbit	Sigma Aldrich	F7425
Monoclonal FK2 (ubiquitin)	Mouse	Enzo Life Sciences	BWL-PW8810
Monoclonal anti-Eps8	Mouse	BD Bioscience	610144
Monoclonal anti-γ-Tubulin	Mouse	Sigma Aldrich	T6557
Monoclonal anti-HGS	Rabbit	Cell Signaling Technology	15087
Monoclonal anti-GSTP1	Mouse	Cell Signaling Technology	3369
Monoclonal anti-β-actin	Mouse	Sigma Aldrich	A1978
Monoclonal anti-Plk1	Rabbit	Cell Signaling Technology	4513
Monoclonal anti-CyclinB1	Mouse	Santa Cruz	sc-245
Polyclonal anti-Arl13b	Rabbit	Proteintech	17711-1-AP
Polyclonal anti-CSRP2	Rabbit	Proteintech	10892-2-AP
Polyclonal anti-Sec23b	Rabbit	Sigma Aldrich	HPA008216
Polyclonal anti-ODF2	Rabbit	Proteintech	12058-1-AP
Polyclonal anti-Fbxw5	Rabbit	[24]	n/a
Polyclonal anti-RanGAP1	Goat	[78]	n/a
Polyclonal anti-Uba2	Goat	[79]	n/a
Anti-Kif2c	Mouse	Santa Cruz	sc-81305
Anti-Kif2c*	Rabbit	Novus Biologicals	NB100-2588
Anti-Kif2a	Rabbit	Abnova	PAB-12407
Anti-Kif2b	Rabbit	Sigma Aldrich	HPA024795
Anti-Mouse-488	Donkey	Invitrogen	A21202
Anti-Mouse-594	Donkey	Invitrogen	A21203
Anti-Rabbit-488	Donkey	Invitrogen	A21206
Anti-Rabbit-594	Donkey	Invitrogen	A21207
**Chemicals, reagents, enzymes & Media**			
**Name**		**Source**	**Identifier**
0.45 µm pore size polyvinylidene difluoride membranes (PVDF)		Amersham	10600021/10600023
aprotinin		Roth	A162.3

ATP	Sigma Aldrich	n/a
Cellfectin-II	Invitrogen (Thermo Fisher Scientific)	10362100
DMEM	Gibco (Thermo Fisher Scientific)	41965-039
DMEM Glutamax^{TM}	Gibco (Thermo Fisher Scientific)	10566016
DMEM-F12	Gibco (Thermo Fisher Scientific)	21331-020
Doxycyclin	Sigma Aldrich	24390-14-5
DYKDDDDK	Pantecs	N/A
Energy regeneration system	Boston Biochem	B-10
ExCell® 420 Serum-Free Medium	Sigma Aldrich	14420C
ExCell® 420 Serum-Free Medium	Sigma Aldrich	14420C
Fetal Bovine Serum (FBS), Qualified	Gibco (Thermo Fisher Scientific)	10270-106
Fluorescence mounting medium	Dako	S3023
HA-peptide (YPYDVPDYA)	Pepscan Therapeutics or Peptides & Elephants	N/A
HiFi assembly mix	New England Biolabs	E2621
Hoechst 33342	Sigma Aldrich	23491-52-3
Human ProtoArray® Microarray (v5.0)	ThermoFischer Scientific/Invitrogen	PAH0525101
Hydroxyurea	Sigma Aldrich	127-07-1
Immobilon Western chemiluminescent HRP substrate	Merck Millipore	WBKLS0500
L-Glutamine 200 mM	Gibco (Thermo Fisher Scientific)	25030-024
Leupeptin Hemisulfat	Roth	CN33.4
Lipofectamin RNAi MAX	Invitrogen (Thermo Fisher Scientific)	13778150
Monoclonal anti-Flag-agarose	Sigma Aldrich	A2220
Monoclonal anti-HA-agarose	Sigma Aldrich	A2095
Ni-NTA-Agarose	Qiagen	30250
Nocodazole	Sigma Aldrich	31430-18-9
OptiMEM	Gibco (Thermo Fisher Scientific)	31985-047
PefaBloc® SC	Roche	11 585 916 001
PepstatinA	Roth	2936.3
Polyethyleneimide (PEI)	PolySciences	23966
Protein A agarose	Roche	11 134 515 001
Protino Glutathione Agarose 4B	Machery & Nagel	745500
Q5 Polymerase	New England Biolabs	M0491
Rabbit IgG isotype control	ThermoFischer Scientific/Invitrogen	10500C

Restriction enzymes		New England Biolabs	-
RO-3306		Sigma Aldrich	SLM0569
Rotiphorese® 30		Roth	3029.1
Sf-900^{TM} II SFM medium		Gibco (Thermo Fisher Scientific)	10902096
Sf-900^{TM} II SFM medium		Gibco (Thermo Fisher Scientific)	# 10902096
Synthetic Block (10x)		ThermoFischer Scientific/Invitrogen	PA017
TEMED		Roth	2367.3
Thymidine		Sigma Aldrich	T9250-1G
β-mercaptoethanol		Applichem	A1108,0025
***E. coli* strains**			
DH5α:	F-φ80lacZ ΔM15 (lacZYA-argF) U169 deoR recA1 endA1 hsdR7 (rk-, mk+) phoA supE44 thi-1 gyrA96 relA1 λ-		
Rosetta:	F- ompT hsdSB (rB- mB-) gal dcm (DE3) pRARE (CmR)		
**Recombinant proteins (expressed and purified from E.coli)**			
**Name**	**Tag**	**Expression plasmid**	**Source**
APPBP1-Uba3	Cleaved	pGEX-GST-APPBP1-rbs-Uba3	This work
Cdc34	Cleaved	pGEX6P3-GST-Cdc34	This work
Kif2a	6xHis	pET28a-His-Kif2A	This work
Kif2b	6xHis	pET28a-His-Kif2B	This work
MCAK	6xHis	pET28a-His-MCAK	This work
Nedd8	Cleaved	pGEX-GST-Nedd8	This work
Uba1	6xHis	pET22B-Uba1-His	This work
Ubc12	Cleaved	pGEX-GST-Ubc12	This work
UbcH5b	Cleaved	pGEX6P3-GST-UbcH5b	This work
UbcH5c	Cleaved	pGEX6P3-GST-UbcH5c	This work
Ube2G1	Cleaved	pGEX6P3-GST-Ube2G1	This work
Ubiquitin	6xHis	pET28a-His-Ubiquitin	This work
Ubiquitin	-	pET3a-Ubiquitin	This work
Ubiquitin (K48R)	-	pET3a-Ubiquitin(K48R)	This work
Ubiquitin (K48R/K63R)	-	pET3a-Ubiquitin(K48R/K63R)	This work
Ubiquitin (K63R)	-	pET3a-Ubiquitin(K63R)	This work
Ubiquitin (methylated)	-	-	Boston Biochem (U-501)
Ubiquitin (FITC-labelled)		Thermo Fisher Scientific	PV4378
Cul1A-Cul1B/Rbx1		-	B. Schulmann
TEV(tobacco etch virus) protease	6xHis	His-TEV	This work

Prescission protease	GST	pGEX-4T-GST-PreScission	This work
**Recombinant proteins (expressed and purified from Sf21 cells)**			
**Name**	**Tag**	**Expression plasmid**	**Source**
Cul1/Rbx1 complex	TEV cleaved	pBIG1B-HisCul1/mmRbx1	This work
Cul4A/DDB1/Rbx1 complex	TEV cleaved	pBIG1B-His-Cul4A/His-DDB1/His-mmRbx1	This work
Eps8	6xHis	pFastBacHT-B-Eps8	This work
Fbxl2/Skp1ΔΔ subcomplex	6xHis	pFastBacHT-B-His-Fbxl2<br>pFastBac1-Skp1ΔΔ	This work
Fbxw5/Skp1ΔΔ subcomplex	6xHis	pFastBacHT-B-His-Fbxw5<br>pFastBac1-Skp1ΔΔ	This work
Fbxw7/Skp1ΔΔ subcomplex	6xHis	pFastBacHT-B-His-Fbxw7<br>pFastBac1-Skp1ΔΔ	This work
Kif2a	TEV cleaved	pFastBacHT-B-His-Kif2A	This work
MCAK	TEV cleaved	pFastBacHT-B-MCAK	This work
Plk1-T210D	6xHis	pFastBacHT-B-His-Plk1-T210D	This work
**siRNAs**			
**Target**	**Sequence**	**Source**	**Reference**
non-targeting (NT)	5'-UAGCGACUAAACACAUCAAUUdTdT-3'	Ambion/Qiagen	[24]
Fbxw5#1	5'-CCACAGGCGCCAAGAGCAAdTdT-3'	Ambion / Sigma Aldrich	[24]
Fbxw5#2	5'-CGGGAGAGGUGGAGAUGCUdTdT-3'	Ambion	[24]
Fbxw5#3	5'-CCCUACAACUGGAGCUACAdTdT-3'	Dharmacon / Sigma Aldrich	[24]
MCAK	5'-GAUCCAACGCAGUAAUGGUdTdT-3'	Sigma Aldrich	[61]
Kif2a	5'-GGAAUGGCAUCCUGUGAAA-3'	Sigma Aldrich	[83]
Kif2b	5'-GGACCUGGAUAUCAUCACC-3'	Sigma Aldrich	[49]

* MCAK antibody NB100-2588 was used for Figure 6B, Figure 7B, EV3A, EV4A,B and F. For all other western blots and all IF images, MCAK antibody sc-81305 was used.
