# Supplementary material for: SCFFbxw5 targets kinesin‐13 proteins to facilitate ciliogenesis
Source: EMBO J. 2021 Aug 9;40(18):e107735. doi: 10.15252/embj.2021107735 (PMC8441365; doi:10.15252/embj.2021107735)
Supplement: Supplementary file 3 — Expanded View Figures PDF [file EMBJ-40-e107735-s005.pdf]

Expanded View Figures

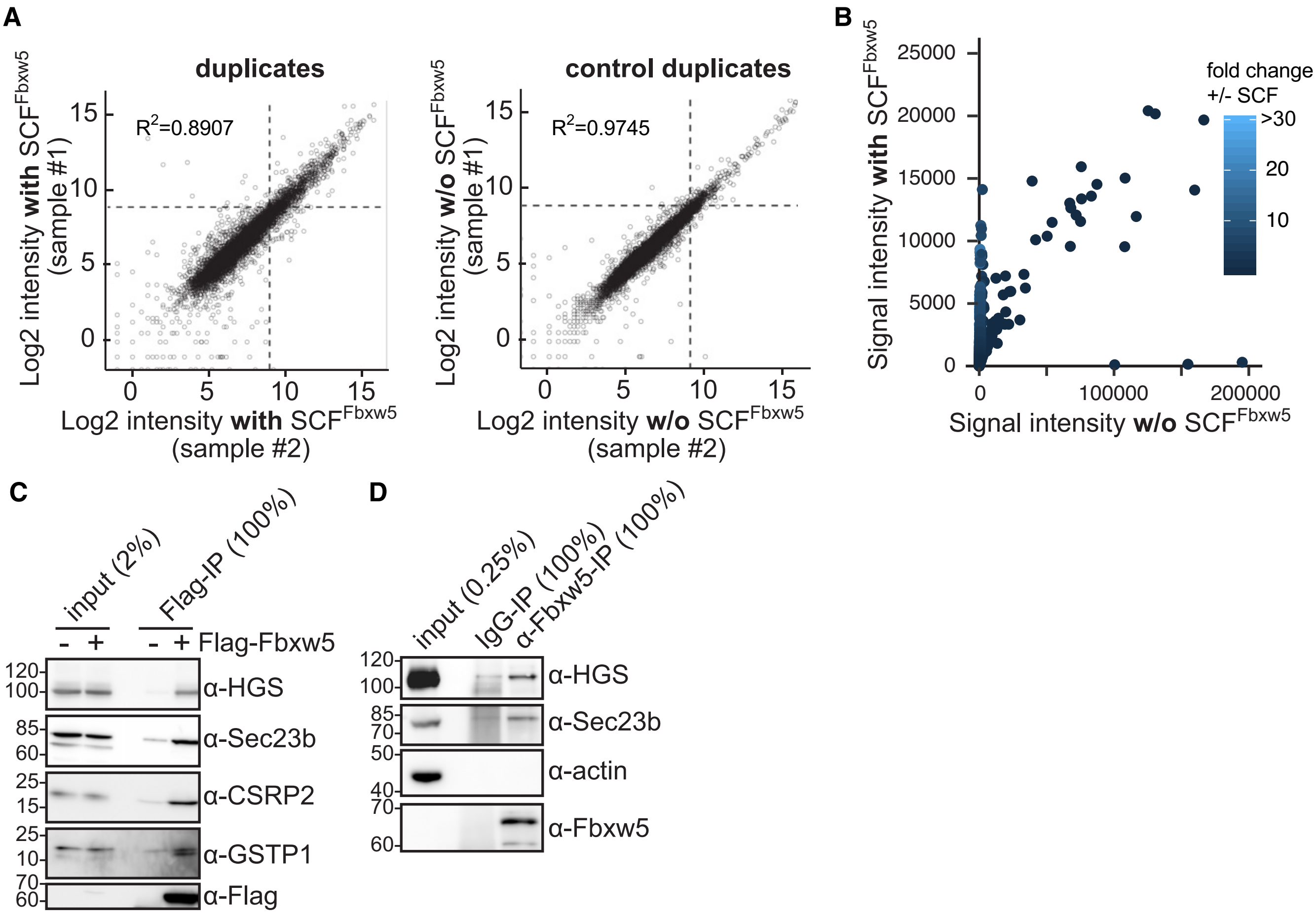

Figure EV1. Further analysis of protein microarray data and candidate substrate validation. Related to Fig 1.

A Scatter plots comparing intensities of duplicate SCF^{Fbxw5} (left) and control (right) samples. Dotted lines indicate the cut-off for candidate substrates (500 AU).

B Comparison of all protoarray signal intensities probed with or without SCF^{Fbxw5} complexes.

C Co-IP of endogenous HGS, Sec23b, CSRP2 and GSTP1 with exogenously expressed Flag-Fbxw5 using anti-Flag antibodies.

D Co-IP of endogenous HGS and Sec23b with endogenous Fbxw5 as in (C), except that anti-Fbxw5 antibodies were used instead of anti-Flag.

Data information: Source data are presented in Dataset EV1.
Source data are available online for this figure.

© 2021 The Authors

Figure EV2. Additional characterisation of kinesin-13 ubiquitylation by Fbxw5. Related to Fig 3.

A Coomassie-stained SDS–PAGE of neddylation enzymes purified from *Escherichia coli* (left) and Cul1/Rbx1 and Cul4A/DDB1/Rbx1 complexes purified from Sf21 cells (right) together with Ube2G1 from *E. coli*. Note that in contrast to Fig 1, Cul1 is purified here as full-length protein and runs therefore as one single band at around 85 kDa.

B Coomassie-stained SDS–PAGE of Cul1 (left) and Cul4A (right) complexes before and after neddylation (N8 = Nedd8).

C *In vitro* ubiquitylation. 0.2 μM MCAK was incubated with 75 μM ubiquitin (untagged or His_6-tagged), 170 nM E1, 1 μM of UbcH5b, 0.15 μM Fbxw5/Skp1 and 0.15 μM Cul1~Nedd8/Rbx1 at 30°C for 45 min followed by Western blotting using anti-MCAK (top).

D Experiment as in (C), except that 0.2 μM His_6-Eps8 was used and incubation time was 90 min at 30°C.

E Experiment as in Fig 3B with Cdc34 as E2, except that untagged, His_6-tagged or Flag-tagged ubiquitin was used.

F Time course experiment as in Fig 3C, except that 0.25 μM UbcH5b was used and reaction was carried out for 90 min at 30°C.

G 0.2 μM MCAK was incubated with 170 nM E1, 1 μM UbcH5c, 1 μM Ube2G1 and 0.2 μM Fbxw5 premixed either with 0.2 μM Cul1~Nedd8/Rbx1 (left) or 0.2 μM Cul4A~Nedd8/DDB1/Rbx1 (right) at 30°C for 15 min.

H Coomassie-stained SDS–PAGE of F-box proteins, each purified from Sf21 cells in complex with Skp1 using same procedures.

I Experiment as in Fig 3E, except that 0.25 μM of UbcH5b was used and reaction was carried out at 30°C for 60 min.

J Comparison of MCAK ubiquitylation purified from *E. coli* or Sf21 cells. Experiment as in Fig 3B, except that for blots 1&2 1 μM of UbcH5b and 0.15 μM SCF^{Fbxw5} were used. For blot 3, 0.25 μM Cdc34 and 0.05 μM SCF^{Fbxw5} were used.

K Coomassie-stained SDS–PAGE of different ubiquitin variants. Methylated ubiquitin (last lane) was obtained from Boston Biochem, and all others were purified from *E. coli*.

L Experiment as in Fig 3G with Cdc34 as E2, except that Kif2a purified from Sf21 cells was used instead of MCAK and reaction was carried out for only 5 min at 30°C.

M Mass spectrometry analysis of lysine residues of MCAK ubiquitylated by SCF^{Fbxw5} and Cdc34 *in vitro*. 4 μM MCAK was incubated with 75 μM ubiquitin, 0.7 μM E1, 1 μM Cdc34, 0.2 μM SCF^{Fbxw5} and 10 mM ATP at 30°C for 15 min. Following SDS–PAGE and in-gel digestion, peptides were analysed by an Orbitrap Q Exactive mass spectrometer. Left: Coomassie-stained gel. Excised regions are indicated. Middle: Cake chart showing identified residues compared to residues annotated at www.phosphosite.org. Right: Cartoon indicating the identified residues. Lysines that are also annotated at phosphosite.org are shown in black and others in grey.

Data information: Source data for M are presented in Dataset EV2.
Source data are available online for this figure.

 © 2021 The Authors

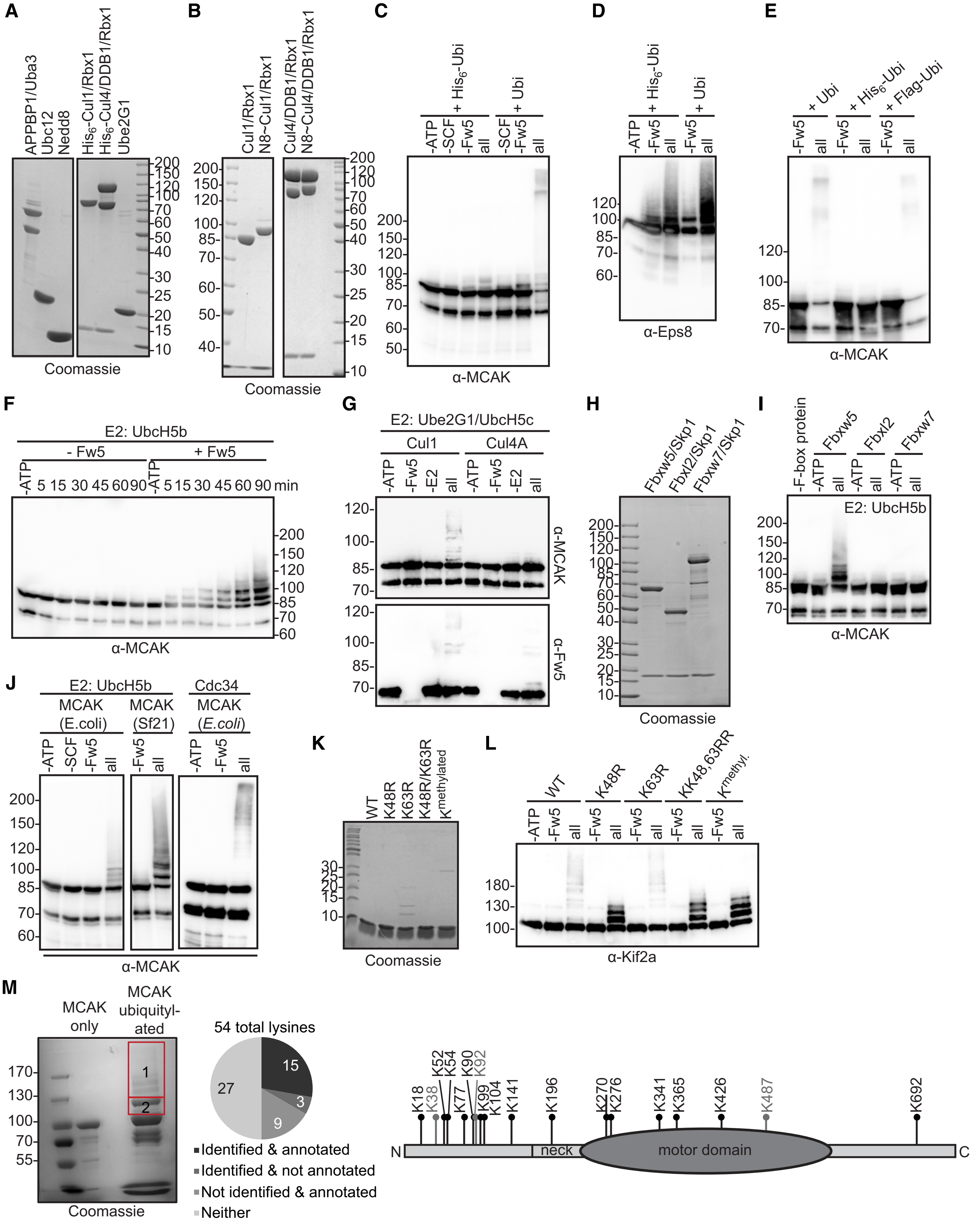
A
APPBP1/Uba3
Ubc12
Nedd8
His6-Cul1/Rbx1
His6-Cul4/DDB1/Rbx1
Ube2G1
Coomassie
B
Cul1/Rbx1
N8~Cul1/Rbx1
Cul4/DDB1/Rbx1
N8~Cul4/DDB1/Rbx1
Coomassie
C
+ His6-Ubi
+ Ubi
-ATP
-SCF
-Fw5
all
α-MCAK
D
+ His6-Ubi
+ Ubi
α-Eps8
E
+ Ubi
+ His6-Ubi
+ Flag-Ubi
α-MCAK
F
E2: UbcH5b
- Fw5
+ Fw5
-ATP 5 15 30 45 60 90 -ATP 5 15 30 45 60 90 min
α-MCAK
G
E2: Ube2G1/UbcH5c
Cul1
Cul4A
-ATP
-Fw5
-E2
all
α-MCAK
α-Fw5
H
Fbxw5/Skp1
Fbxl2/Skp1
Fbxw7/Skp1
Coomassie
I
-F-box protein
Fbxw5
Fbxl2
Fbxw7
E2: UbcH5b
α-MCAK
J
E2: UbcH5b
Cdc34
MCAK (E.coli)
MCAK (Sf21)
MCAK (E.coli)
α-MCAK
K
WT
K48R
K63R
K48R/K63R
K methylated
Coomassie
L
WT
K48R
K63R
KK48,63RR
K methyl.
α-Kif2a
M
MCAK only
MCAK ubiquityl-ated
1
2
Coomassie
54 total lysines
15
3
9
27
Identified & annotated
Identified & not annotated
Not identified & annotated
Neither
K18 K38 K52 K54 K77 K90 K92 K99 K104 K141 K196 K270 K276 K341 K365 K426 K487 K692
N
neck
motor domain
C

Figure EV2.

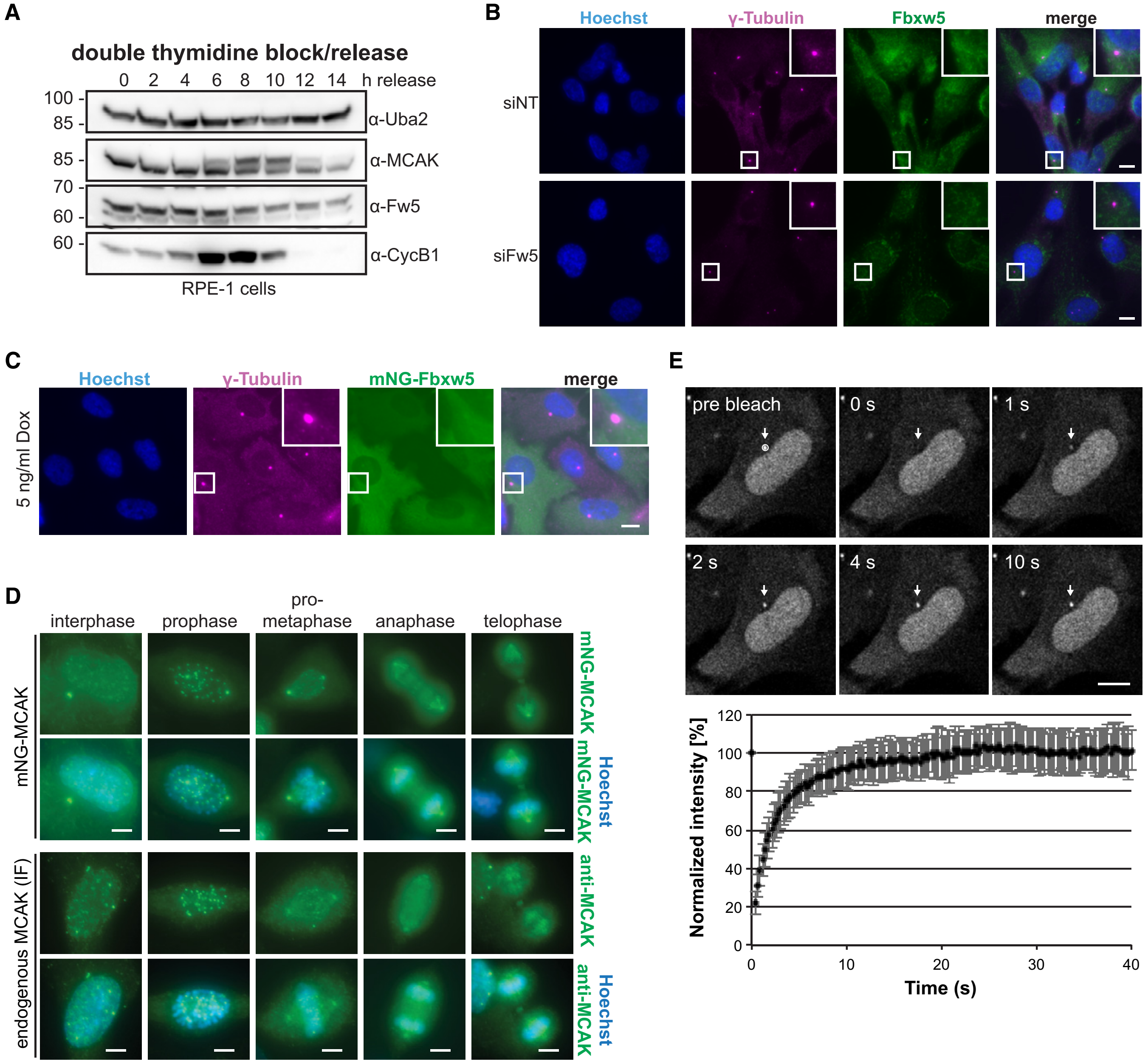

Figure EV3. Characterisation of Fbxw5 localisation and MCAK dynamics at centrosomes. Related to Fig 4.

A Double thymidine block/release experiment. Cells were arrested with 200 mM thymidine for 16 h, released for 9 h and again arrested for 16 h. After a second release, cells were harvested at the indicated time points and analysed by Western blotting using the indicated antibodies for detection.

B IF of RPE-1 cells treated with the indicated siRNAs for 48 h using the indicated antibodies for detection together with Hoechst staining. Scale bar = 10 µm.

C IF of RPE-1 cells stably expressing mNG-tagged Fbxw5 under a doxycycline-inducible promoter. Cells were induced with 5 ng/ml doxycycline for 24 h, and IF was performed using the indicated antibody for detection together with Hoechst staining. Scale bar = 10 µm.

D Comparison of mNG-MCAK localisation with endogenous MCAK by IF. Top panels show mNG-MCAK signals of methanol-fixed and Hoechst-stained cells in different cell cycle stages. Bottom panel shows endogenous MCAK signals obtained by IF with anti-MCAK antibodies in methanol-fixed and Hoechst-stained wild-type RPE-1 cells. Scale bar = 5 µm. Note that different min/max settings have been applied for better visualisation.

E FRAP analysis of polyclonal RPE-1 cells expressing mNG-MCAK induced with 5 ng/ml doxycycline for 24 h seeded on Ibidi 8 Well Glass Bottom µ-slides. Top: Representative FRAP experiment with images before (pre-bleach) and after photobleaching (0 s) of the area outlined (white circle) and at later times (upper left corners). The recovery area is indicated (white arrows). Scale bar = 10 µm. See also Movie EV1. Bottom: Mean recovery curve over 40 s upon photobleaching of the centrosome signal at 0 s. Error bars show standard deviation of 26 FRAP-analysed cells.

Data information: Source data for E are presented in Source Data for Fig EV3.
Source data are available online for this figure.

© 2021 The Authors

Figure EV4. Fbxw5 knockdown affects MCAK levels already before mitotic exit. Related to Fig 5.

A Confirmation of specificity for rabbit MCAK antibody (NB100-2588). RPE-1 cells were treated with the indicated siRNAs and analysed by Western blotting.

B Immunoblot of monoclonal RPE-1 cell line used in Fig 5A expressing mNG-MCAK under a doxycycline-inducible promoter, treated with the indicated siRNAs for 48 h and induced with 6 ng/ml doxycycline for the last 24 h.

C IF of cells used in (B), treated with DMSO or 6 ng/ml doxycycline for 24 h and stained with Hoechst and MCAK antibody to compare signals of endogenous with overexpressed MCAK. Scale bar = 10 μm.

D RPE-1 cells were treated with the indicated siRNAs for 48 h and arrested with 75 ng/ml nocodazole for 5 h. Mitotic cells were shaken off, washed twice with PBS, plated on coverslips in serum-free medium and fixed at the indicated time points with methanol followed by IF using the indicated antibodies together with Hoechst staining. For 0-h time point, cells were directly seeded on coverslips after siRNA treatment and harvested after 5 h of nocodazole arrest. Left: Representative images. Arrowheads indicate ODF2 signal. Scale bar = 5 μm. Note that for 0-h time point, different min/max display settings were applied as for the other time points for better visualisation (settings are always kept the same within each time point, and no adjustment was applied for quantification). Right: Quantification. For time point 0 h, all ROIs (obtained by thresholding) were considered (kinetochore signals). For time points 4, 18 and 24 h, only signals co-localising with the ODF2 spot were considered (centrosomal signals). Error bars show standard error of the mean of three independent experiments covering in total more than 150 cells. Asterisks indicate *P*-value of a Mann–Whitney test comparing Fbxw5 knockdown with control samples for each time point (***$P < 0.001$).

E Polyclonal RPE-1 cells were treated with the indicated siRNA for 24 h, split on Ibidi 8 Well Glass Bottom μ-slides and mNG-MCAK expression was induced with 10 ng/ml doxycycline for another 24 h. Cells in prometaphase were imaged using spinning disk microscopy. Left: Representative images. ROI used for measuring signal intensities is indicated (dashed circle). Scale bar = 5 μm. Right: Quantification of total mNG-MCAK signal intensity in prometaphase cells of three independent experiments each covering more than 30 cells. Error bars indicate standard error of the mean and asterisks the *P*-value of a two-tailed unpaired Student's *t*-test (***$P < 0.001$).

F Left: Polyclonal RPE-1 cells expressing mNG-MCAK under a doxycycline-inducible promoter were induced with 10 ng/ml doxycycline for 24 h and analysed by IF using Hoechst staining and MCAK antibody to compare signals of endogenous with overexpressed MCAK. Scale bar = 10 μm. Right: Same cells treated with different amounts of doxycycline (as indicated, 0 = DMSO control) for 24 h and analysed by Western blotting.

Data information: Source data for D are presented in Source Data for Fig EV4.
Source data are available online for this figure.

© 2021 The Authors

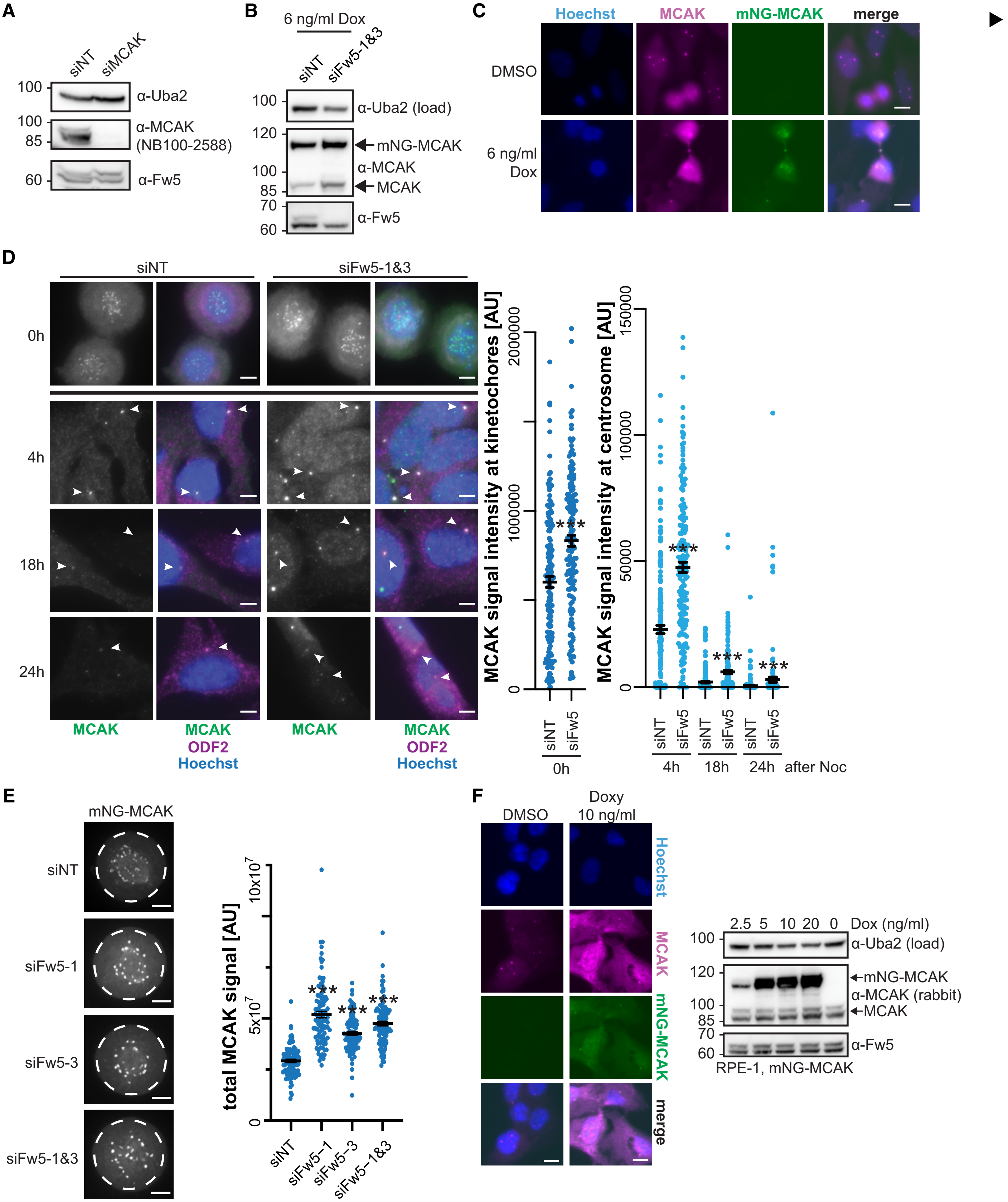

Figure EV4.

 © 2021 The Authors

Figure EV5. Kinesin-13 proteins are involved in the Fbxw5-dependent ciliogenesis defect. Related to Fig 7.

A Polyclonal RPE-1 cells stably expressing H2B-mRuby2 under a CMV promoter were transfected with the indicated siRNAs for 48 h and then imaged using Incucyte® S3. Chart shows the mean percentage of mitotic cells and error bars the standard deviation of three experiments, each covering more than 2,500 cells.

B Quantification of the length of remaining cilia of RPE-1 cells upon mNG-MCAK overexpression (same samples as in Fig 7A). Long black line shows mean intensity, error bars show standard error of the mean of five independent experiments covering in total more than 150 cilia, and asterisks indicate *P*-value of a two-tailed unpaired Student's *t*-test (***$P < 0.001$).

C Correlation between MCAK signal intensity and cilia length of same samples as in (B). MCAK signal intensity was measured within a circular ROI at basal bodies with a diameter of 1.65 μm and plotted against the according length of the remaining cilia. Solid line shows simple linear regression and dashed line the 95% confidence bands. R^2 and the *P*-value of a Pearson correlation analysis are shown.

D Quantification of the length of remaining cilia of RPE-1 cells upon siRNA treatment (same samples as in Fig 7C). Long black line shows mean intensity, error bars show standard error of the mean of four independent experiments each covering more than 30 cilia, and asterisks indicate *P*-value of a two-tailed unpaired Student's *t*-test comparing the indicated samples (***$P < 0.001$).

E Extracts of RPE-1 cells treated with the indicated siRNAs for 72 h and serum-starved for the last 24 h analysed by Western blotting. Left: Representative blot. Right: Quantification of Kif2a/Uba2 signal ratio. Error bars show standard deviation of five independent experiments and asterisks the *P*-value of a two-tailed unpaired Student's *t*-test (**$P < 0.01$ and ***$P < 0.001$).

F RPE-1 cells stably expressing mNG-Kif2b under a doxycycline-inducible promoter were treated with the indicated siRNA for 72 h. 24 h after siRNA transfection, mNG-Kif2b expression was induced with 10 ng/ml doxycycline. 24 h later, doxycycline was washed out and cells were serum-starved for another 24 h followed by Western blot analysis with the indicated antibodies. Left: Representative blots. Right: Quantification of mNG-Kif2b/Uba2 signal ratio. Error bars show standard deviation of five independent experiments and asterisks the *P*-value of a two-tailed unpaired Student's *t*-test (**$P < 0.01$).

G Extracts of RPE-1 cells treated with the indicated siRNAs for 72 h and serum-starved for the last 24 h followed by Western blot analysis. Left: Representative blot. Right: Quantification of Kif2a/Uba2 signal ratio. Error bars show standard deviation of four independent experiments and asterisks the *P*-value of a two-tailed unpaired Student's *t*-test comparing each knockout with the non-targeting control (*$P < 0.05$ and ***$P < 0.001$).

H IF of RPE-1 cells treated with the indicated siRNAs for 72 h and serum-starved for the last 24 h using the indicated antibodies together with Hoechst staining. Left: Representative images. Scale bar 10 μm. Right: Quantification of ciliated cells. Error bars show standard deviation of three independent experiments each covering more than 200 cells and asterisks the *P*-value of a two-tailed unpaired Student's *t*-test (*$P < 0.05$, **$P < 0.01$ and ***$P < 0.001$).

Data information: Source data for (A, B, C, D, E, F, G, H) are presented in Source Data for Fig EV5.
Source data are available online for this figure.

© 2021 The Authors

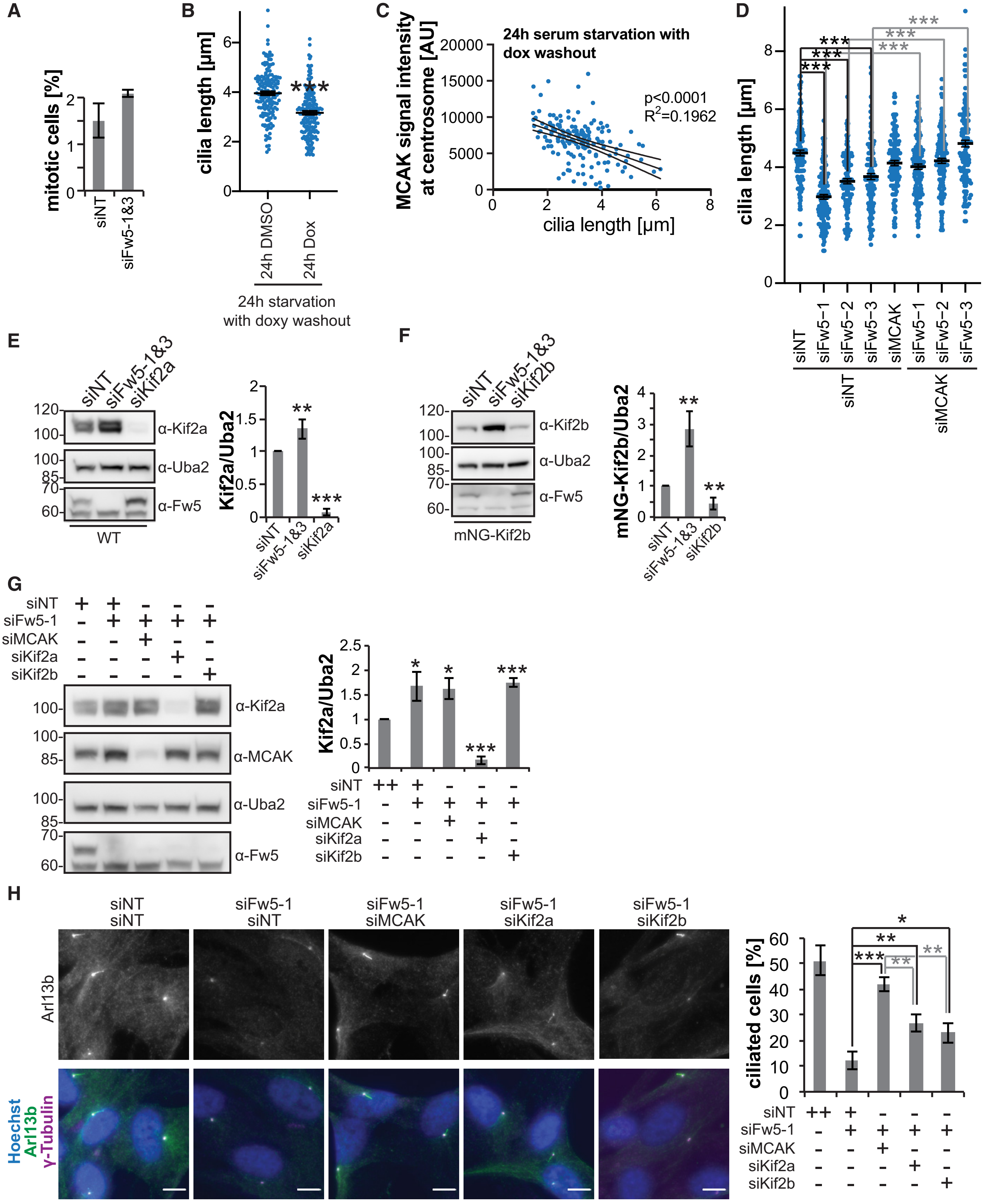

Figure EV5.

 © 2021 The Authors
