# Supplementary material for: SCFFbxw5 targets kinesin‐13 proteins to facilitate ciliogenesis
Source: EMBO J. 2021 Aug 9;40(18):e107735. doi: 10.15252/embj.2021107735 (PMC8441365; doi:10.15252/embj.2021107735)
Supplement: Supplementary file 6 — Movie EV1 [file EMBJ-40-e107735-s004.zip › Movie EV1.docx]

# Movie EV1

Representative time-lapse movie of Fig EV3E. Please refer to Source Data for Fig EV3E for exact time points. Scale bar = 10 µm
