# Supplementary material for: SCFFbxw5 targets kinesin‐13 proteins to facilitate ciliogenesis
Source: EMBO J. 2021 Aug 9;40(18):e107735. doi: 10.15252/embj.2021107735 (PMC8441365; doi:10.15252/embj.2021107735)
Supplement: Supplementary file 7 — Movie EV2 [file EMBJ-40-e107735-s006.zip › Movie EV2.docx]

# Movie EV2

Representative time-lapse movie of Fig 5A with one image every 20 min. Left: non-targeting control. Right: Fbxw5 knockdown cell. Scale bar 10 µm.
