# Supplementary figures and images for: SCFFbxw5 targets kinesin‐13 proteins to facilitate ciliogenesis
Source: EMBO J. 2021 Aug 9;40(18):e107735. doi: 10.15252/embj.2021107735 (PMC8441365; doi:10.15252/embj.2021107735)

Source Data for Figure EV1 (whole scans of blots)

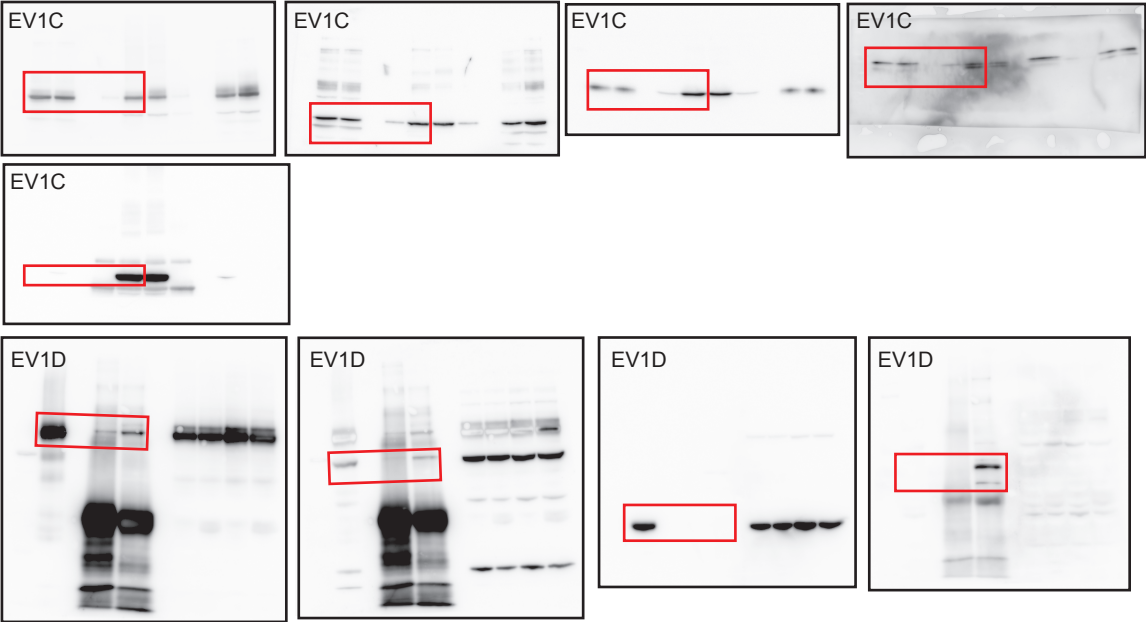

Supplement: Supplementary file 8 — Source Data for Expanded View [file EMBJ-40-e107735-s015.zip › EMBOJ-2021-107735R1-Figure_EV1_Source_Data-sd.pdf]

Source Data for Figure EV2 (whole scans of gels/blots)

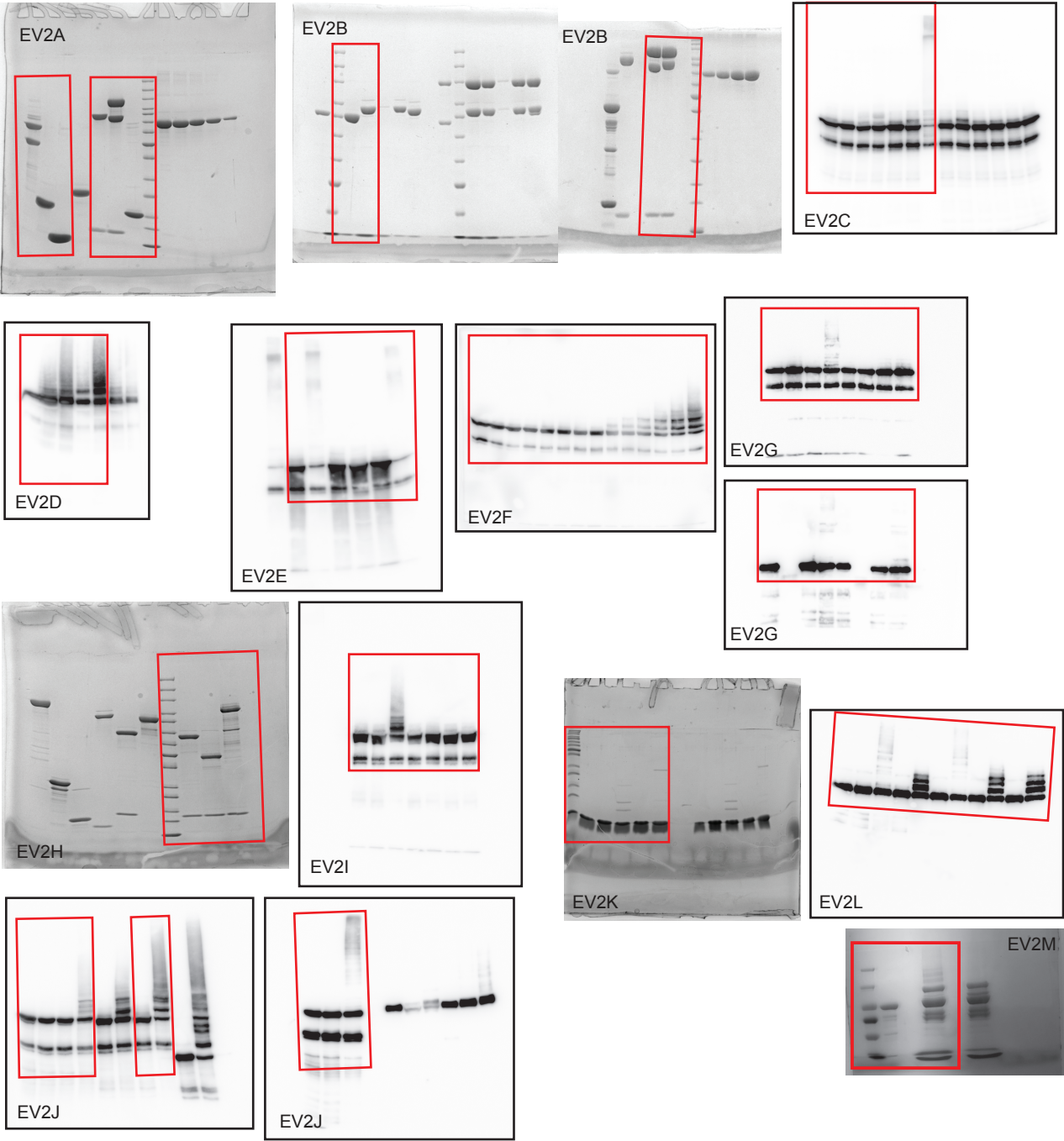

Supplement: Supplementary file 8 — Source Data for Expanded View [file EMBJ-40-e107735-s015.zip › EMBOJ-2021-107735R1-Figure_EV2_Source_Data-sd.pdf]

Source data for Figure 1 (whole scans of blots)

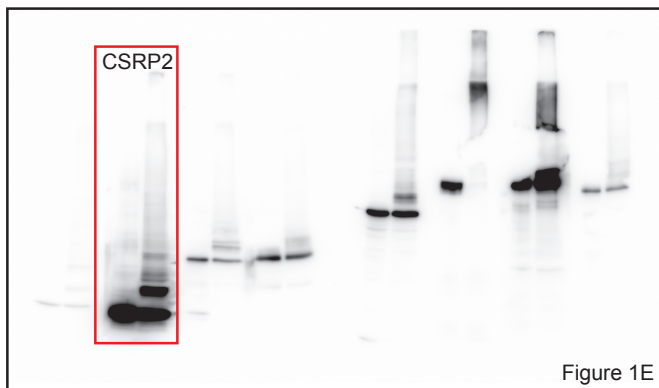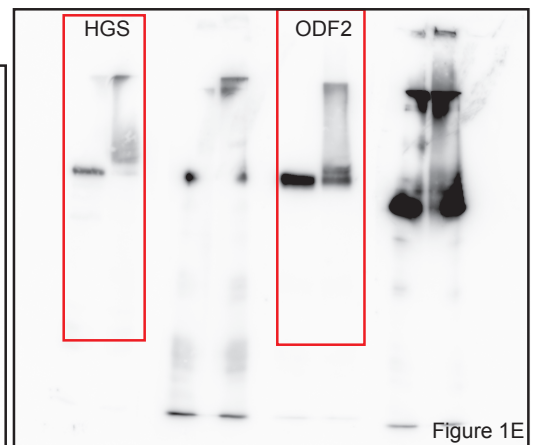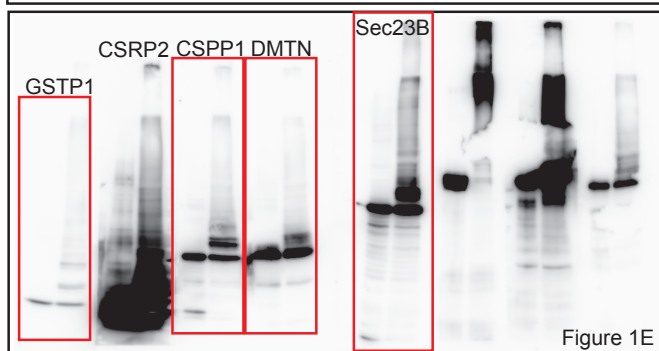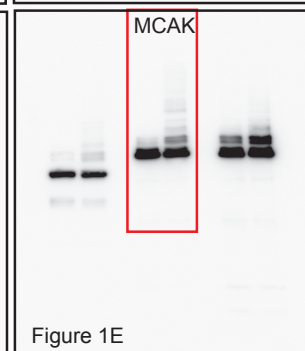

Supplement: Supplementary file 9 — Source Data for Figure 1 [file EMBJ-40-e107735-s008.pdf]

Source data for Figure 2 (whole scans of gels/blots)

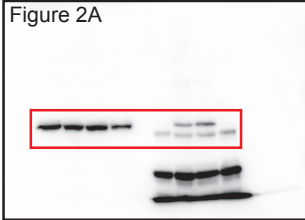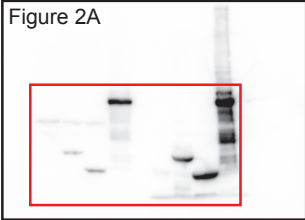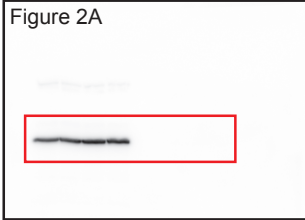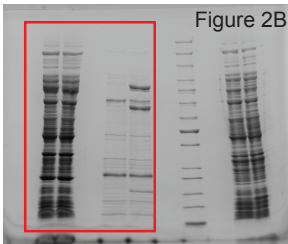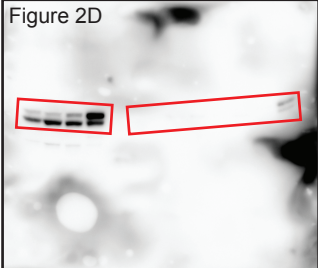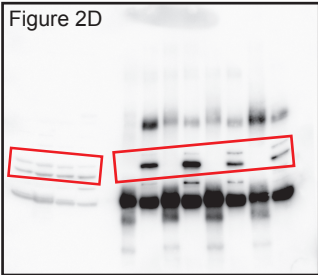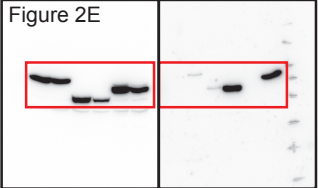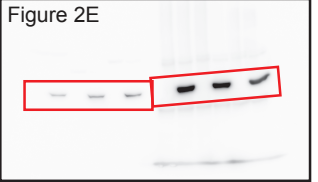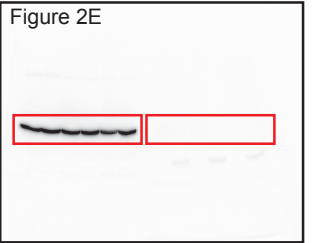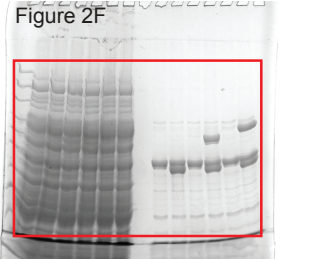

Supplement: Supplementary file 10 — Source Data for Figure 2 [file EMBJ-40-e107735-s002.zip › Source Data for Figure 2.pdf]

Source data for Figure 3 (whole scans of gels/blots)

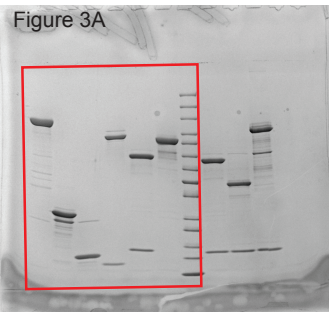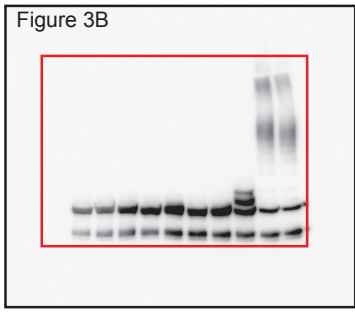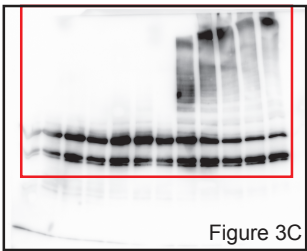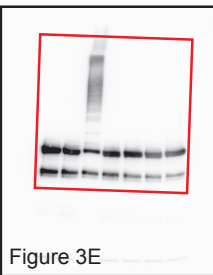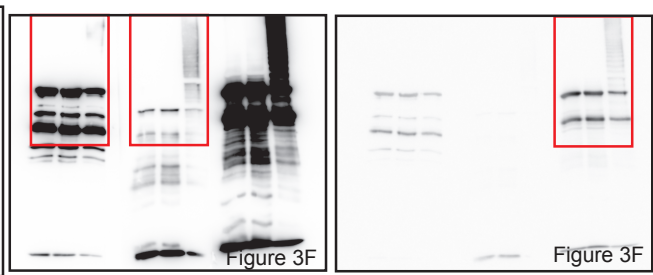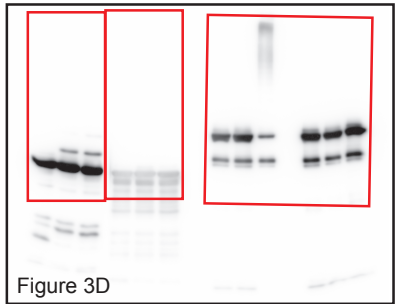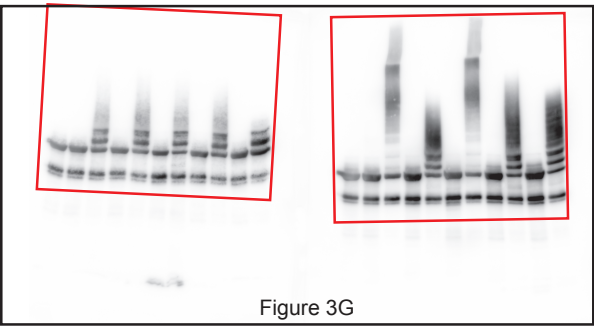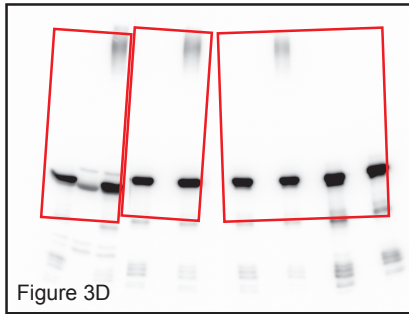

Supplement: Supplementary file 11 — Source Data for Figure 3 [file EMBJ-40-e107735-s009.pdf]

Source data for Figure 4 (whole scans of blots)

Figure 4A

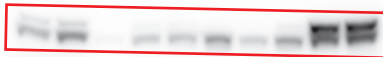

Figure 4A

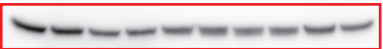

Figure 4A

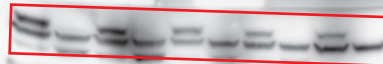

Supplement: Supplementary file 12 — Source Data for Figure 4 [file EMBJ-40-e107735-s010.zip › Source Data for Figure 4.pdf]

Source data for Figure 5 (whole scans of blots)

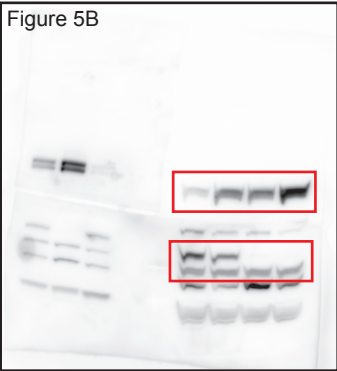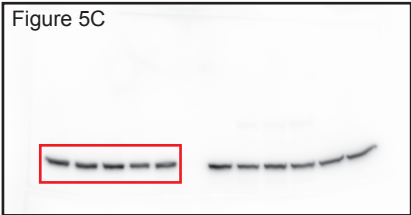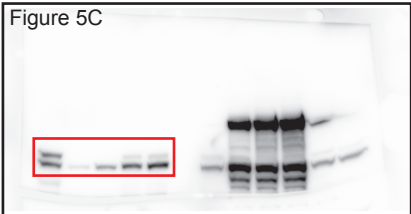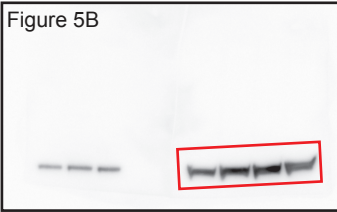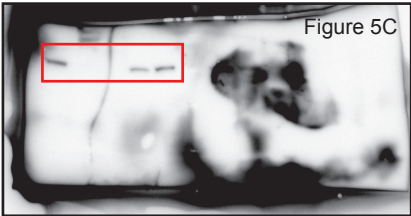

Supplement: Supplementary file 13 — Source Data for Figure 5 [file EMBJ-40-e107735-s003.zip › Source Data for Figure 5.pdf]

### Source Data for Figure 6 (whole scans of blots)

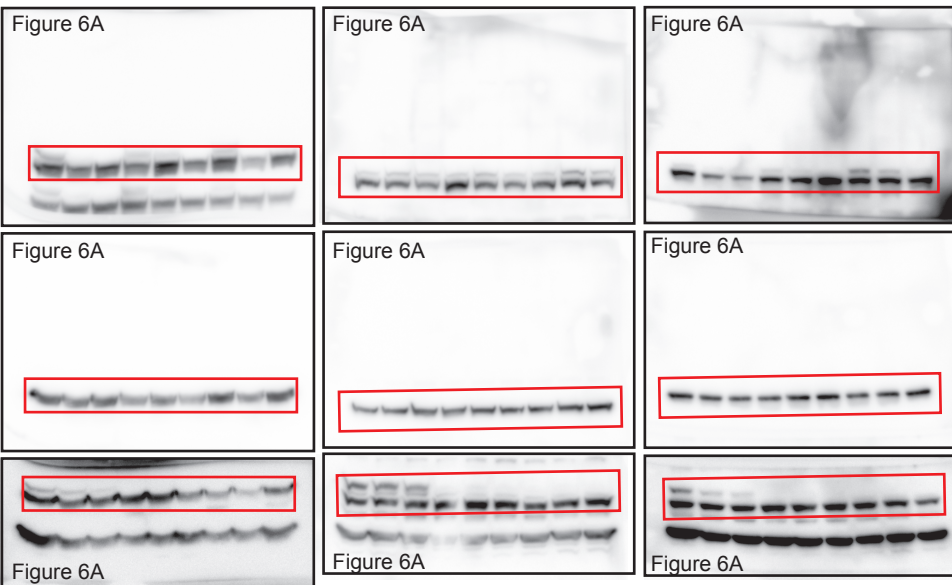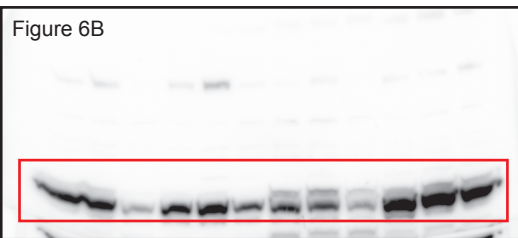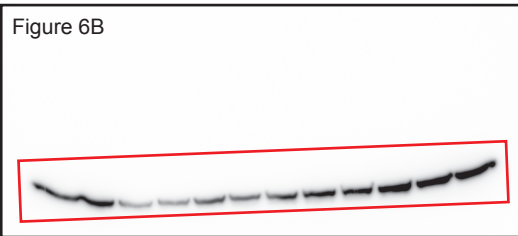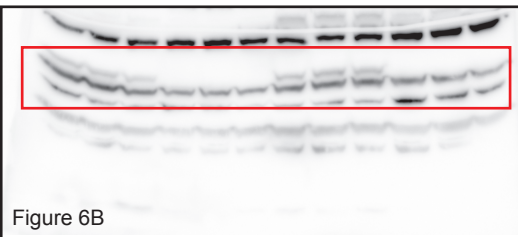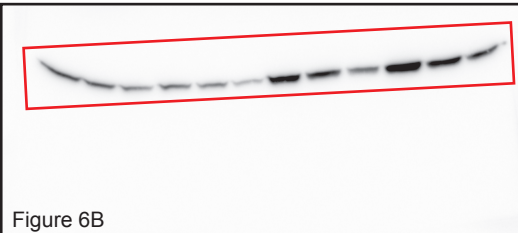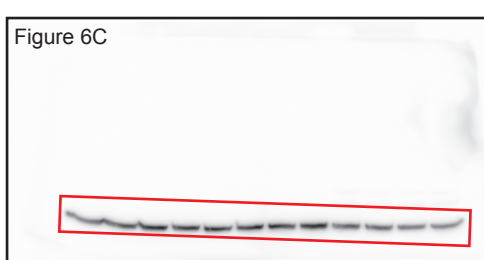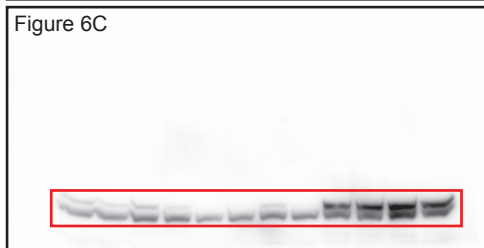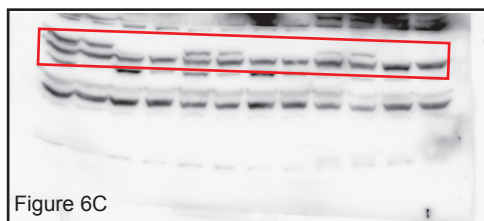

Supplement: Supplementary file 14 — Source Data for Figure 6 [file EMBJ-40-e107735-s014.zip › Source Data for Figure 6.pdf]

Source Data for Figure 7 (whole scans of blots)

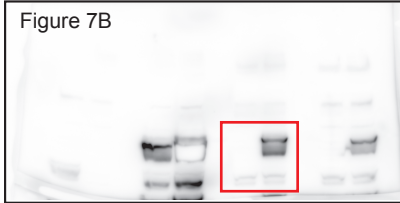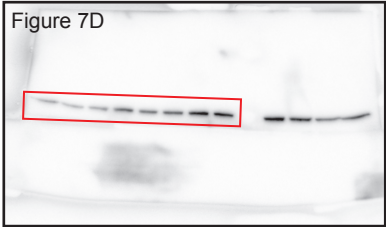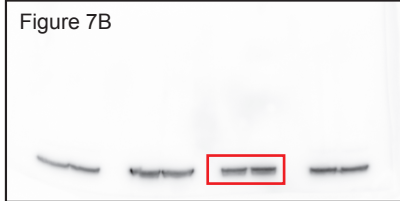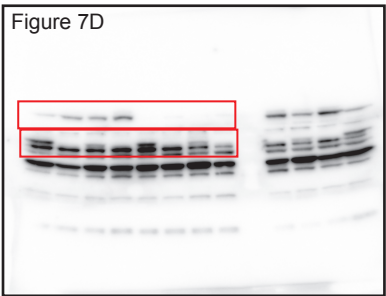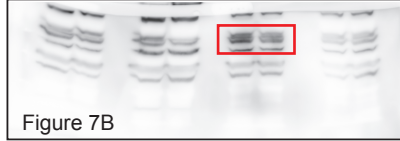

Supplement: Supplementary file 15 — Source Data for Figure 7 [file EMBJ-40-e107735-s012.zip › Source Data for Figure 7.pdf]
