# Supplementary material for: SCFFbxw5 targets kinesin‐13 proteins to facilitate ciliogenesis
Source: EMBO J. 2021 Aug 9;40(18):e107735. doi: 10.15252/embj.2021107735 (PMC8441365; doi:10.15252/embj.2021107735)

Source Data for Figure EV5 (whole scans of blots)

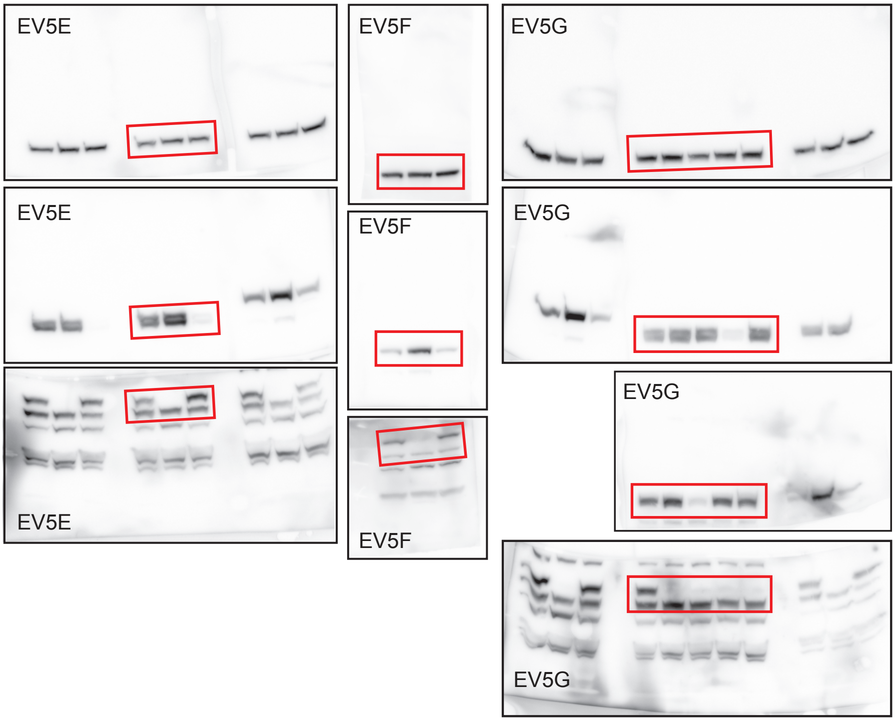

Supplement: Supplementary file 8 — Source Data for Expanded View [file EMBJ-40-e107735-s015.zip › EMBOJ-2021-107735R1-Figure_EV5_Source_Data-sd(1).zip!/Source Data for Figure EV5.pdf]
